# Editorial: Invertebrate Models of Natural and Drug-Sensitive Reward

**DOI:** 10.3389/fphys.2019.00490

**Published:** 2019-04-26

**Authors:** Moira van Staaden, Robert Huber

**Affiliations:** J.P. Scott Center for Neuroscience, Mind & Behavior, Bowling Green State University, Bowling Green, OH, United States

**Keywords:** drug addiction, invertebrate, psychostimulant, review, alcohol

The relationship between insects and humans is a complex one, characterized biotically as commensalism, mutualism, or parasitism. This collection of papers reveals yet another dimension, in which shared history invests invertebrate models with the power to interrogate critical challenges to the human reward system. As humans we take pride in approaching a given scenario rationally, in considering the possible options, assigning them values, and then choosing that which maximizes one's individual outcomes. So, why do drug addicts make choices that inevitably lead to ruinous consequences? Fundamentally, addiction appears to impair the very ability to form considered judgments, as it strips the afflicted of this most essential of human faculties. What are *Homo sapiens* bereft of their “sapient” power? Despite widespread recognition of the devastating and lasting effects of addiction, there is little consensus regarding its mechanistic and perceptual causes, or on effective therapeutic interventions. Policy makers, healthcare specialists, and the general public, frequently view drug dependence as an incompetent life choice, or moral failure resulting from a fundamental lack of willpower. However, the moralistic perspective falls short in generating effective solutions. It provides little help to the addicted, offers no support to those in the addict's immediate social circle, and gives no guidance in addressing the significant and growing societal burden posed by substance abuse (Florence et al., 2016). In contrast, the US National Institute of Drug Abuse has strongly advocated for a brain disease model of addiction (BDMA), and empirical findings in behavioral neuroscience have advanced promising avenues for reframing this phenomenon from a more structural perspective (Volkow and Koob, 2015). Viewed as a chronic, relapsing brain disorder, addiction is characterized as a dysregulation of reward, motivation, judgment and memory, with associated changes in neuronal structure and function, and where medical technologies offer the clearest path to treatment. A third, more holistic perspective, dissents rather forcefully from the medical cure inherent in the BDMA approach (Lewis, 2015). In this view, addiction results from the development of all-consuming patterns of conduct in which initially formed mental and behavioral habits become self-perpetuating. Liberation from the yoke of addiction thus will come from engraving new beneficial patterns over the existing harmful ones, and will depend crucially on a conducive social environment. Regardless of specific distinctions, all of these explanatory models share the fundamental assumption that addiction resides squarely within the cognitive domain.

A unifying view emerging from this compilation of drug-sensitive reward in invertebrates suggests that we may do well to critically evaluate fundamental notions about addiction vulnerabilities and the causes of compulsive drug taking. First, we ought to recognize addiction as a phenomenon with exceedingly deep evolutionary roots. Though commonly referred to as “human drugs of abuse,” addictive plant alkaloids emerged as potent chemical defenses against insect herbivory (Wink). The Achilles heel of animals then, resides in the tradeoffs required for efficient learning–balancing specificity with generalization, and computational speed with behavioral flexibility. Shaped by an evolutionary arms race, compounds such as nicotine, cathinone, and morphine, evolved to interfere with essential functions of learning in their insect pests. Acting as weaponized disruptors of conserved learning machinery, they commandeer neural drivers for motivated search and dysregulate the circuits for reward perception. The very nature of this interference limits the evolution of effective countermeasures. For instance, mutations lessening an individual's sensitivity to these defenses inevitably incur significant side effects of reduced initiative, lowered reward perceptions, and critically impaired decision-making. Within this broader context, addicted humans represent collateral damage arising from the homology of ancestral learning mechanisms tracing back to the early divergence of bilateral metazoa. Aside from fetal drug exposure or postoperative analgesics, initial drug consumption is generally a choice and not an evolutionary imperative. However, once addictive alkaloids have compromised the basic learning circuitry, higher-order executive functions (including cognition and willpower) have limited authority against them.

Second, we should acknowledge that these deep evolutionary roots imbue invertebrate models with unique power to reveal the origin and development of reward system function. The papers gathered in this volume demonstrate the rich spectrum of behavioral and neural consequences exhibited by invertebrate preparations in response to drug exposure. These addiction-like phenomena parallel the full range of effects identified in mammalian models ranging from activational responses associated with common psychostimulants, sensitization on repeated application, extinction, withdrawal, and reinstatement. Drug self-administration, considered the most valid experimental model for drug-seeking and -taking behaviors, and the final step in preclinical testing of potential treatments, is observed in roundworms self-exposure to cocaine, nicotine and methamphetamine (Engleman et al.), and in crayfish self-administering amphetamine (Datta et al.). Invertebrate models also exhibit strong tendencies for relapse after extended periods of abstinence. Søvik et al. employ a proboscis extension reflex learning paradigm to explore whether cocaine affects memory processing independently of its effect on incentive salience in honey bees. Their finding that cocaine strongly impairs consolidation of extinction memory is key to understanding how cocaine exerts enduring impacts on behavior.

We underscore the benefits of broad taxonomic inclusion for illuminating aspects of addiction that are of clinical importance, but for which few suitable animal models exist. For instance, Lee et al. describe spontaneous amphetamine-induced escape swims in a sea slug, *Tritonia diomedea*, triggered by false perceptions of predator contact (i.e., hallucinations). Unconditioned exposure to mammalian drugs of abuse generate a variety of stereotyped behaviors in crayfish, stimulating exploration and appetitive motor patterns along with molecular processes for drug conditioned reward in novel contexts (Shipley et al.). Step-wise alteration of the phenotype in taxa with incomplete metamorphosis permits longitudinal analysis of behavioral states with a degree of precision that is otherwise difficult to achieve.

Third, since natural reward is a life-sustaining process central to learning, comparison with drug-sensitive reward can identify the genetic and neural mechanisms underlying appetitive reward, and most urgently, the factors contributing to addiction vulnerabilities. What variation promotes the progression from initial drug use to addiction? Important insights come from contributions in *Drosophila*, a model system with powerful genetic tools and a vibrant research community. With an emphasis on how motivational states shape the value of the rewarding experience, Ryvkin et al. modeled different aspects of natural and drug rewards. They conclude that it is social isolation, pain, deprivation and stress that shape the repertoire/function of proteins and mediate reward processing. Lowenstein and Velazquez-Ulloa establish the functional modularity of reward circuits in *Drosophila* resembles that of mammals.

Aminergic systems in associative learning transmit prediction error signals and convey stimulus prediction signals for the execution of conditioned responses. Mizunami and Matsumoto review work in crickets, honey bees, and fruit-flies, demonstrating the conserved nature of reward systems in insects and mammals, along with diversity in the neurotransmitters mediating appetitive signaling. Elucidating the computational rules underlying such activity at the molecular level requires a combination of functional and behavioral analyses. Lanzo et al. used cell-specific knockdown *in vivo*, and cultured neurons *in vitro*, to demonstrate that the dopamine transporter in the cell membrane of *C. elegans* functions primarily to reuptake dopamine from the synaptic cleft back into the dopaminergic neurons, an activity altered by drugs of abuse such as amphetamine.

A major challenge to the discovery of effective addiction therapies is our limited understanding of underlying mechanisms and of potential therapeutic targets. One promising approach aims to assess comparative reward strength of individual drugs using operant, self-administration or conditioned place preference paradigms, with the high-throughputs made possible by invertebrates. Engleman et al. propose just such a system, presenting data for *C. elegans* with predictive validity as a behavioral medication screen.

Alcohol Use Disorder is a major health, social and economic problem with few effective treatments. Four studies illustrate invertebrate contributions at levels from molecular to complex memory phenotypes. *Drosophila* develop a preference for ethanol, an effect that is reversed by the opiate antagonist naltrexone (Koyyada et al.). Guevara et al. examined developmental alcohol exposure on feeding behavior in *Drosophila*, where NPF signaling plays a critical role in alcohol-reduced food intake for both larvae and adults. Neural and molecular mechanisms underlying persistent memories of intoxication induce cravings and trigger relapse in recovering individuals. The work of Nunez et al. characterizes the dose-dependent nature of ethanol on the memory expression of intoxication experience, highlighting the advantage of sophisticated behavioral analysis. Fruit flies possess both acute and persistent memories for ethanol-conditioned odor cues, and the state of intoxication influences the retention and expression of associated memories. Finally, Swierzbinski and Heberholz combine electrophysiological and neuropharmacological techniques to exploit the occurrence of two escape mechanisms in crayfish. Their findings suggest intriguing effects of alcohol on the GABAergic system.

The invertebrate taxa reported on in this volume trace the origins of addiction-like phenomena to at least 950 MYA (Dohrmann and Wörheide, 2017). Placozoa occupy a basal position in the metazoan phylogeny and lack a nervous system. The recent demonstration that they coordinate sophisticated behavioral sequences, such as feeding, using an intercellular peptidergic signaling system (Varoqueaux et al., 2018) is the clearest indication yet that the revelatory power of invertebrate models for understanding the intricacies of natural- and drug-sensitive reward has only just begun.

## Author Contributions

All authors listed have made a substantial, direct and intellectual contribution to the work, and approved it for publication.

### Conflict of Interest Statement

The authors declare that the research was conducted in the absence of any commercial or financial relationships that could be construed as a potential conflict of interest.
